# When should social service referral be considered in phenylketonuria?

**DOI:** 10.1016/j.ymgmr.2015.01.002

**Published:** 2015-02-09

**Authors:** Margreet van Rijn, Kirsten Ahring, Amaya Bélanger-Quintana, Kathi Dokoupil, Hulya Gokmen Ozel, Anna Maria Lammardo, Martine Robert, Júlio C. Rocha, Anita MacDonald

**Affiliations:** a Section of Metabolic Diseases, University of Groningen, University Medical Centre Groningen, Groningen, The Netherlands; b Department of Clinical Genetics, Copenhagen University Hospital, Kennedy Centre, Glostrup, Denmark; c Unidad de Enfermedades Metabolicas, Hospital Ramon y Cajal, Madrid, Spain; d Department of Metabolism and Nutrition, Dr von Hauner Children's Hospital, University of Munich, Munich, Germany; e Faculty of Health Sciences, Department of Nutrition and Dietetics, Hacettepe University, Ankara, Turkey; f San Paolo Hospital, University of Milan, Milan, Italy; g Nutrition and Metabolism Unit, Hôpital Universitaire des Enfants Reine Fabiola, Brussels, Belgium; h Centro de Genética Médica Doutor Jacinto de Magalhães, CHP EPE, Porto, Portugal; i Faculdade de Ciências da Saúde, Universidade Fernando Pessoa, Porto, Portugal; j Center for Health Technology and Services Research (CINTESIS), Porto, Portugal; k The Children's Hospital, Birmingham, United Kingdom

**Keywords:** HCPs, healthcare professionals, Phe, phenylalanine, PKU, phenylketonuria., Phenylketonuria, Adherence, Social services, Child protection, Foster care, Case study

## Abstract

Lifelong low-phenylalanine (Phe) dietary management is the foundation of care in phenylketonuria (PKU). However, strict monitoring of food intake places a burden on patients and their caregivers, and adherence to the required diet frequently decreases in later childhood and adolescence. Rarely, parents of children with PKU refuse to recognise the importance of treatment and follow-up for this chronic condition. Here, two case studies are presented that document consideration of placement of children into foster care or kinship homes as a last resort to improve persistently high Phe concentrations. In the first case, social service referral led to a 3-year-old girl being placed in a kinship home with her grandparents, resulting in excellent Phe control thereafter. In the second case, discussion with the parents of possible placement of a 12-year-old child into foster care was sufficient to have a positive effect on Phe control. A staged approach for managing intractable non-adherence in PKU is proposed.

## 1 Introduction

Phenylketonuria (PKU) treatment mainly involves a strict, lifelong, low-phenylalanine (Phe) diet, commenced in the newborn period. This regimen poses a clear challenge for patients and their parents/caregivers, and treatment adherence and long-term Phe control may be poor [1], [2], [3], [4], leading to suboptimal clinical outcomes in school age children [5]. Associations between poor quality blood Phe control and behaviour problems, sustained attention and lower IQ are well documented [6], [7]. Healthcare workers seek to optimise adherence through intensive dietary management training for patients and parents, and regular patient monitoring and follow-up. However, the capacity of patients and parents to follow a treatment plan is influenced by factors such as family, social and economic status, patient's psychological status, severity of the treatment regimen, and local treatment standards and protocols. Cases in which parents of a child with PKU persistently refuse to engage with healthcare professionals (HCPs) and support essential treatment requirements are a particular concern. Engagement of social/child protective services for possible transfer of the child into a kinship home or foster care may be considered in extreme cases. This is a very strong measure, and when and how it should be implemented, and its benefit, remain uncertain. In PKU, there are no agreed criteria to define extreme non-adherence and no directional guidance for HCPs considering such a course of action to ensure that a consistent approach is adopted and understood by parents, patient support groups and HCPs.

In this paper, we describe two case studies in which failure of parental adherence with low-Phe diet led to referral to social services by the treating HCP. Both patients were diagnosed with PKU by newborn screening, prescribed with low-Phe diet treatment since early infancy, and followed by an expert treatment centre. Caregivers gave written consent.

## 2 Case study 1

A 2-year-old girl with classical PKU had only 43% of her blood Phe concentrations within target range (120 to 360 μmol/L) over the first two years of life. The child was cared for by a young single mother without higher education, the administration of Phe-free l-amino acid supplements was inconsistent, dietary Phe was unmeasured and mealtimes were unsupervised. A hospital admission (aged 2 years) was associated with immediate improvement in blood Phe control, but there was deterioration post-discharge. The clinical care team was confident that the mother understood the diet. However, she was inaccessible by telephone, clinic attendance worsened and blood samples were infrequent. Between 2 and 3 years of age, only 2% of blood Phe concentrations were within target range. The mother received extensive practical help (low-protein cooking, nursery placement, collection of blood samples and organisation of dietary prescriptions). The mother rejected teaching of the extended family. Social services intervention led to no further improvement in dietary control, but within 6 months, a court of law decreed the child stay with maternal grandparents in a ‘kinship’ placement. Excellent blood Phe control was subsequently maintained for > 7 years with > 80% of blood Phe concentrations within target range. At the age of 11 years the patient had not had a formal IQ test; she attended normal school and was in the top quartile of her class year for many subjects, including English and literature, but she found mathematics difficult and had poor organisational skills.

The same hospital PKU standards of management and PKU care were applied pre- and post-‘kinship placement’: weekly blood Phe samples taken by caregivers, clinic attendance every 3 months and blood Phe concentrations maintained between 120 μmol/L and 360 μmol/L. The PKU team considered 70% or more of the blood Phe concentrations within target range to be acceptable control. If more than 50% of the phe levels were above the target range, the PKU team would institute extra education for the child's family. This included low-protein cooking classes and support worker home visiting.

## 3 Case study 2

A 12-year-old girl with classical PKU had a long history of unacceptable control with only 15% of blood Phe concentrations < 600 μmol/L the previous year. She had a young mother, a stepfather, and lived on a remote island some distance from the PKU centre. The parents provided a variety of reasons for poor adherence to treatment, including illness, dislike and inadequate supply of Phe-free l-amino acids and school bullying. Despite intensive practical help and support of a psychologist, metabolic control did not improve. Blood Phe monitoring became less regular, and the family became less accessible. The PKU centre finally referred her to social services. They suggested possible child removal if adherence did not improve.

In the 5 months prior to social service involvement, blood Phe concentrations had ranged from 252 μmol/L to 1448 μmol/L (mean 791 μmol/L). This was well above the target of < 600 μmol/L.

In the 3 months following social service involvement, blood Phe ranged from 201 μmol/L to 784 μmol/L (mean 538 μmol/L). The frequency of home blood Phe sampling had also improved. Over the 2 years following social service involvement, 74% of blood Phe concentrations were within target range, and 90% of blood Phe samples were returned. The mother became more responsive, answering hospital phone calls and engaging appropriately with HCPs. She ordered Phe-free l-amino acid supplement as prescribed, which the girl took without persuasion. An IQ test performed 8 months post-social service involvement indicated the overall performance was at the lower end of the normal range.

## 4 Discussion

It is essential that therapy in PKU is adhered to given well-documented evidence of poor neurocognitive and psychological outcomes in patients with off-target Phe levels [8], [9], [10].

These are the first case studies in PKU to describe extreme parental non-adherence to dietary treatment leading to referral to social services and/or transfer of a child to a kinship home. In both cases involvement of social services had a positive clinical impact. These cases provide a springboard for discussion on a measure that some HCPs view as controversial.

A number of common factors led ultimately to the engagement of social services in these cases including: persistently poor Phe levels in early life; failure to return regular blood samples for Phe tests and foster regular contact/visits to the clinic; and poor parental attitude to treatment, as evidenced by failure of standard educational approaches to improve outcomes. Features of these cases might also suggest ‘risk factors’ for chronically poor adherence, including young and single parent/s, lack of parental higher education [11], and lack of extended family support. Other known risk factors include alcoholism, drug abuse and mental health problems; it is known in the UK that between 50% and 90% of parents of children on social workers' child care caseloads have one or more of these issues, affecting their parenting capacity [12].

Foster or kinship care should remain a last resort following failure of all other approaches [13], as it is associated with child behavioural issues and poor mental health [14], [15]. The final decision as to whether to remove a child from his or her parents is generally a legal one, and the level of proof that child welfare is at risk generally has to be high [16]. From a HCP's perspective, the challenge is if and when to raise the alarm, and this responsibility should not be taken lightly; the UK Government takes the view that HCPs are in a strong position to identify welfare needs or safeguarding concerns regarding individual children and, where appropriate, provide support’ [17], and further warns that ‘no professional should assume that someone else will pass on information which they think may be critical to keeping a child safe’. In the US, mandated ‘reporter’ laws exist, which may legally oblige treating physicians to contact child protective services in certain instances of apparent parental neglect [18]. Governments and/or health authorities in most countries issue guidelines for HCPs to report and provide evidence in cases of suspected child neglect (e.g. see reference [17]).

However, even when alerting social/child protective services is warranted, some HCPs working with patients with PKU may be reluctant to pursue this course of action. As PKU is a lifelong condition, a key goal of HCPs is to establish close and trusting long-term relationships with the families they are involved with, and calling social/child protective services under any circumstances could be viewed as a violation of this trust. Additional pressures may include uncertainty regarding the level of parental neglect, fear that foster/kinship care might not improve Phe control and/or lead to other comorbidities, fear of subsequent legal action from families, and even concerns about the time demands for additional long-term statutory safeguarding meetings and vigilant documentation. The medical literature on social service engagement in PKU is sparse, which provides little in the way of reassurances to HCPs. The frequency with which parents blatantly disregard dietary management in PKU is unknown, and the long-term outcome of a legal care order demanding child transfer from parental care to a kinship or foster family is not documented in PKU.

There has been recent debate in the US on the intervention of social/child protective services in severe cases of childhood obesity, which provides an interesting basis for discussion among PKU professionals. A framework for the identification of child obesity cases for referral to social/child protective services has been presented [19], and it has been suggested that in severe instances of childhood obesity linked to chronic parental neglect, removal from the home may be legally justifiable [18]. Furthermore, medical evidence has been presented for a benefit of foster homing in three patients with extreme childhood obesity [20], and in a US-based cohort of children entering into foster care, the frequency of obesity decreased over 1 year in care [21]. Counter arguments for intervention cite uncertainties regarding the level of parental neglect [22], [23], lack of imminent danger to child welfare [23], concerns regarding the validity of suggested social intervention criteria [19], [24], and the complex legalities and consequences of transferring a child into a foster home [16], [22]. The same arguments could be applied in the realm of PKU.

To avoid inconsistent practices, and provide reassurances to all stakeholders, evidence-based guidelines or expert consensus may be warranted for cases of chronically poor parental adherence to the PKU dietary management of their child. Several key aspects would need to be covered to provide a framework for a methodical and staged approach (Table 1[17], [25], [26], [27], [28]).

**Table 1:** Management considerations for non-adherence in PKU.

Management considerations		Details
Definition of non-adherence		Agree upon a definition of unacceptable non-adherence in PKU • E.g., number of blood Phe concentrations outside target range within a defined time-period
Early warning signs that adherence may be a concern		• Failure to attend clinic appointments • Lack of blood samples • Unresponsive to telephone calls • Not co-operative with nursery/school staff or other agencies • Consistently runs out of l-amino acid supplement/low-protein special foods because of poor organisation • Poor metabolic control
Staged management approach in identified cases	1. Education	Provide an education package for parents/caregivers and extended family • E.g., basics of diet, consequences of PKU, importance of blood sample monitoring, and practical advice on low-protein cooking
	2. Treatment	Explore all treatment options • E.g., different types of low-protein supplements with different tastes/conveniences, and tetrahydrobiopterin treatment (sapropterin dihydrochloride [25], [26]) which may help to liberalise diet while controlling Phe levels [27], [28]
	3. Practical support	Engage co-ordinated practical help via inter-agency working (e. [17]) • E.g., involving other HCPs, early years support, and contracted/voluntary services from the hospital and community
	4. Psychology assessment	Refer the caregiver/child for psychology assessment and support
	5. Hospital admission	Admit the child to hospital to prove acceptable Phe concentrations are attainable
	6. Social service involvement	Refer the case to social/child protective services

HCP, healthcare professional; Phe, phenylalanine; PKU, phenylketonuria.

Although HCPs should strive to improve blood Phe control in all age groups of children, there may be more benefit to long-term IQ through earlier intervention. It is established that blood Phe control rarely improves if it has been unacceptable in the first 3 years of life [11], and children with PKU are likely to gain most benefit from social service intervention in pre-school years. Therefore, early warning signs of potential issues with long-term blood Phe control described above should be quickly addressed by working closely with families and other agencies.

In conclusion, HCPs have a responsibility to protect and safeguard children in their care. Ultimately, when parents of a child with PKU choose to blatantly disregard the treatment needs of their child, considering the likely severity of health outcomes, this should be considered as a form of child neglect and therefore within the remit of social/child protective service consideration [19]. In PKU, urgent consensus guidelines are required in this important area to enable HCPs to work within a consistent and supported framework.

## Conflict of interest statement

All authors have received compensation from Merck Serono as members of the European Nutritionist Expert Panel in PKU. Margreet van Rijn is additionally a member of the ELEMENT (Leading Education in Metabolic Error Nutritional Therapy) Steering Committee for Nutricia International, and has received grants and fees for educational and research activities from Nutricia International and Orphan Europe.

Anita MacDonald has received research funding and honoraria from Nutricia, Vitaflo International and Merck Serono. She is a member of the European Nutrition Expert Panel (Merck Serono international), member of Sapropterin Advisory Board (Merck Serono international), and member of the Advisory Board ELEMENT (Danone-Nutricia).

## Funding statement

Neil Burton of Fishawack Communications GmbH provided editorial assistance, which was funded by Merck Serono.
